# Quality Improvement Project on Split-Post Placement in Core Psychiatry Training

**DOI:** 10.1192/bjo.2025.10362

**Published:** 2025-06-20

**Authors:** Hala Elhardlu, Faquiha Muhammad, Suneetha Siddabattuni

**Affiliations:** 1 Northamptonshire NHS Foundation Trust, Northampton, United Kingdom; 2 Lincolnshire NHS Foundation Trust, Lincoln, United Kingdom

## Abstract

**Aims:** Split-post placements are part of Psychiatry training, being a combination of inpatient and outpatient settings. The outpatient post could be set within the community mental health teams outpatient clinics, Crisis teams, Gender Identity and CAMHS clinics.

Trainees in such split-posts typically spend 2–3 days per week doing outpatient work, with the remainder in inpatient settings. The allocation is primarily a factor of training needs, to ensure safe delivery of clinical services, patient safety and provision of appropriate experience. Post allocation ensures trainees have the opportunity to achieve training competencies. This means that while individual preferences cannot always be met, the posts allocated will meet the trainee’s needs.

Our survey consisted of measuring the level of satisfaction with clinical experiences and supervision whilst working in split-posts, and factors pertaining to Trainees’ perception of patient safety, continuity of care and workloads.

**Methods:** Taking into consideration HEE guidelines regarding training placements, we collaborated with trainee programme director and created a qualitative survey including East Midlands Psychiatric Core trainees at Northamptonshire Healthcare Foundation NHS Trust working in split-posts. Of 15 trainees, 9 responded and completed the survey.

**Results:** While our survey respondents were able to identify that split-posts allow for more variety in clinical experience, they also noted several difficulties in transitioning between outpatient and inpatient settings, including:

Inability to keep up with pending work.

Difficulty establishing strong professional relationships with both staff and patients in both settings, as they are only present for 1–2 days.

Interruption in continuity of care, with work from both posts frequently overlapping.

Compromise in the level of supervision available to them, as they were only assigned a clinical supervisor in one setting.

62.5% of trainees found the workload across both placements manageable. However, half of the trainees faced challenges transitioning between clinic and inpatient roles. 37.5% of trainees did not feel adequately supervised in split-posts.

**Conclusion:** Our survey shows room for improvement within split-post placements. Based on our findings, we can advise the following measures to mitigate some of the difficulties for trainees in such posts:

We have created a standardised handover sheet which is advised to be used and updated routinely, so that patient safety and continuity of care is maintained.

We suggest to assign a clinical supervisor to each post within a split-post placement, to ensure a trainee has ease of access to their weekly supervision in either setting, outside of the usual daily clinical discussions.

